# Monitoring of polar organic compounds in fresh waters using the Chemcatcher passive sampler

**DOI:** 10.1016/j.mex.2023.102054

**Published:** 2023-02-03

**Authors:** Rosamund F.A. Robinson, Graham A. Mills, Gary R. Fones

**Affiliations:** a School of the Environment, Geography and Geosciences, University of Portsmouth, Burnaby Road, Portsmouth, PO1 3QL, UK; b School of Pharmacy and Biomedical Sciences, University of Portsmouth, White Swan Road, Portsmouth, PO1 2DT, UK

**Keywords:** Pesticides, Pharmaceuticals, Personal care products, Passive sampling, Rivers, Streams, Chemcatcher, Eu water framework directive (WFD), Environmental monitoring, Monitoring of polar organic compounds using Chemcatcher

## Abstract

The monitoring of polar organic pollutants in surface water is now undertaken to fulfil a number of legislative requirements. Passive sampling is being frequently used for this purpose and includes the commercially available Chemcatcher device. This protocol is based on knowledge that has been acquired over the past ten years in the use of the Chemcatcher for monitoring a wide range of polar organic compounds in freshwater. It provides detailed procedures and guidelines of how to prepare the sampler in the laboratory, deploy and retrieve the device in the field (including water and sampling site measurements) and subsequent sample processing in the laboratory up to instrumental analysis. By end users adopting this standardized, systematic protocol it will help to ensure the reproducibility of their monitoring data.

• Robust and detailed procedure for the sampling of polar pollutants in surface waters using the Chemcatcher passive sampler
• A low cost, novel and versatile apparatus for deploying the Chemcatcher at riverine sites
• Practical tips based on extensive experience of using the Chemcatcher are provided for end-users

## Graphical abstract

**Figure:**
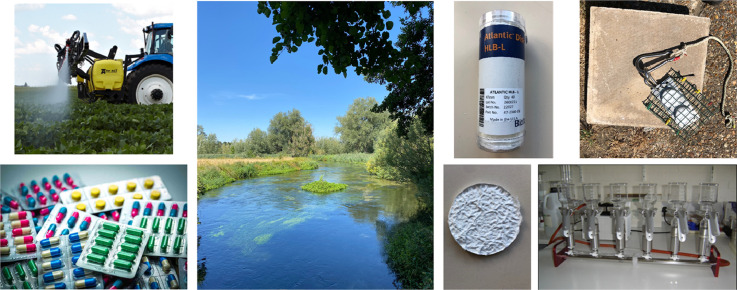

Specifications table

**Table:** 

Subject area	Environmental Science

More specific subject area	Water Quality Monitoring by Passive Sampling
Name of your method	Monitoring of polar organic compounds using Chemcatcher
Name and reference of original method	ISO 5667–23:2011. Water quality –Sampling - Part 23: Guidance on passive sampling in surface waters.
Resource availability	Chemcatcher passive sampler bodies available from T.E Laboratories Ltd, Ireland. https://chemcatcher.ie/ Chemcatcher stainless steel deployment plate available from AT Engineering, Tadley, UK. https://www.atengineeringtadley.co.uk/water-quality-monitoring/

## Method details

### Background

Regular sampling of surface water is required by the Water Framework Directive (WFD; European Directive 2000/60/EC) and its subsequent Daughter Directives [1]. This is to assess whether human activities have any impact on water quality and to assess the effect of contaminants on aquatic biota. The principal method used is discrete (bottle or spot) sampling which provides a snapshot of the contaminants present at the time of collection. Concentrations of pollutants in surface water vary over time and hence it may be more desirable to monitor them over longer time periods. This is to gain a better time-integrated understanding of how stochastic inputs of pollutants affect water quality. Increasing temporal resolution will lead to a better understanding of how chemicals can affect biota. This can be achieved by repeated spot sampling, continuous monitoring, biomonitoring or passive sampling [2].

Passive sampling involves the deployment of a device in the water column over an extended period of time (days to months) where pollutants are continuously sequestered [3,4]. A range of devices is available for monitoring different classes (e.g. non-polar organics, polar organics, metals, nutrients) of pollutants. The Chemcatcher passive sampler has been successfully and frequently used as a monitoring tool for the measurement of polar organic contaminants in surface, waste waters and drinking water [5], [6], [7], [8], [9], [10], [11]. This version of the Chemcatcher comprises a two-component polytetrafluoroethylene (PTFE) body that houses a hydrophilic/lipophilic balanced media (HLB) receiving phase disk overlaid by a polyethersulfone (PES) diffusion membrane.

The protocol described in this article addresses the use of this device for monitoring polar organic compounds in fresh waters. The procedure will give improved guidance to end users of the device and includes details of step by step preparation, deployment, retrieval and extraction procedures up to the point of instrumental analysis. The guidance given expands on the generic passive sampler procedures given in ISO 5667–23:2011 [12].

### Reagents and materials

#### Reagents

• Water, ultrapure (>18.0 MΩ.cm @ 25 °C) (UPW)
• Decon™ 90 (or similar phosphate and chlorine free laboratory detergent)
• Methanol (MeOH) CH_3_OH (HPLC grade)
• Acetone (Propanone) C_3_H_6_O (HPLC grade)

#### Materials

##### Chemcatcher

**Fig. 1:**
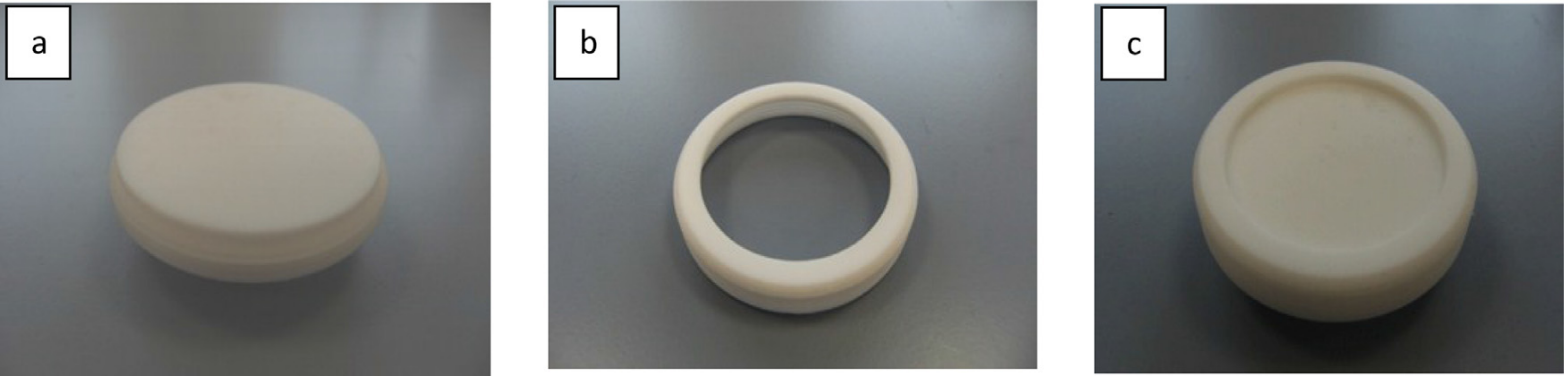
Components of the Chemcatcher device; (a) base plate (b) threaded retaining ring (c) assembled sampler.

Chemcatcher passive sampler bodies (two-component) are available from T.E Laboratories Ltd, Ireland. (https://chemcatcher.ie/) (Fig. 1). They comprise a polytetrafluoroethylene (PTFE) base plate (Fig. 1a) and a threaded retaining ring (Fig. 1b) which are then screwed together (Fig. 1c).

##### Receiving phase disk

**Fig. 2:**
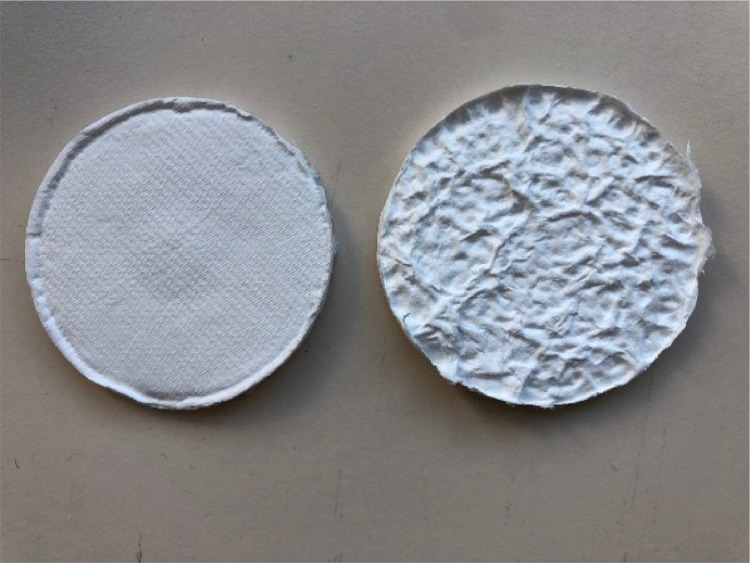
Biotage HLB-L 47 mm diameter receiving disk, showing smooth and rough sides.

The most commonly used receiving phase chemistry for monitoring polar organic compounds is HLB (hydrophilic/lipophilic balanced media). This is available as a bound disk (47 mm diameter) from Affinisep (https://www.affinisep.com/products/spe-disks/attractspedisks-environment/) and Biotage (Fig. 2) (https://www.biotage.com/solid-phase-extraction-disks). For this protocol the use of the Biotage HLB-L disk is described.

#### Diffusion membrane

Polyethersulfone (PES) (Supor® 200, 0.2 μm pore diameter) diffusion membrane.

Available from Pall Europe Ltd, Portsmouth, UK

#### Laboratory equipment

Gloves, powder-free vinyl or powder-free latex

Metal punch (52 mm diameter)

Mallett

Wooden board

Vacuum pump

Extraction manifold and funnel (Available at Affinisep; Biotage; Fisher Scientific and Merck Millipore as single, triple or sextuple versions)

Vacuum pump trap or Büchner vacuum pump flask, installed between the manifold and the vacuum pump to collect liquids that are aspirated and to prevent contamination of the vacuum pump

Sealable polyethylene terephthalate (PET) boxes

Stainless steel tweezers

Glass beakers

Measuring cylinders

#### Deployment and retrieval equipment

Concrete paving slab (400 mm x 400 mm 28 mm) (or similar weighing approximately 10 kg)

Eye bolt, washers, and nuts

Stainless steel deployment plate (180 mm x 80 mm) (available from AT Engineering,

Tadley, UK. [https://www.atengineeringtadley.co.uk/water-quality-monitoring/])

Stainless-steel carabiners (assorted sizes)

Stainless-steel R clips

Plastic coated mesh (200 mm x 160 mm, with 25 mm square mesh openings)

Cable ties

Sealable polyethylene terephthalate (PET) boxes (for transport of Chemcatcher devices)

Cool box and ice packs

Scissors or tin snips

Plastic bags

Labels

Aluminium foil

Telescopic boat hook

#### Generic

Independent or multiparameter probes for in situ measurement of conductivity, depth, dissolved oxygen, pH and temperature.

Digital flow metre (m *s* ^−^ ^1^)

Surveyors tape measure (∼30 m) and water depth pole

#### Procedure

This section provides the procedures for (A) Chemcatcher cleaning and preparation; (B) Chemcatcher deployment and retrieval; (C) water and sampling site measurements; and (D) Chemcatcher receiving phase disk extraction (up to instrumental analysis)

### Chemcatcher cleaning and preparation

• In order to avoid contamination, Chemcatcher preparation and processing should be performed in a clean environment suitable for trace organic analysis
• Use either powder-free vinyl or powder-free latex gloves whilst working in the laboratory or field
• Acetone rinsed stainless-steel tweezers are used to handle the components

#### Cleaning Chemcatcher housing

Before assembling the Chemcatcher, all PTFE components must be washed using a four-step procedure:

**Figure:**
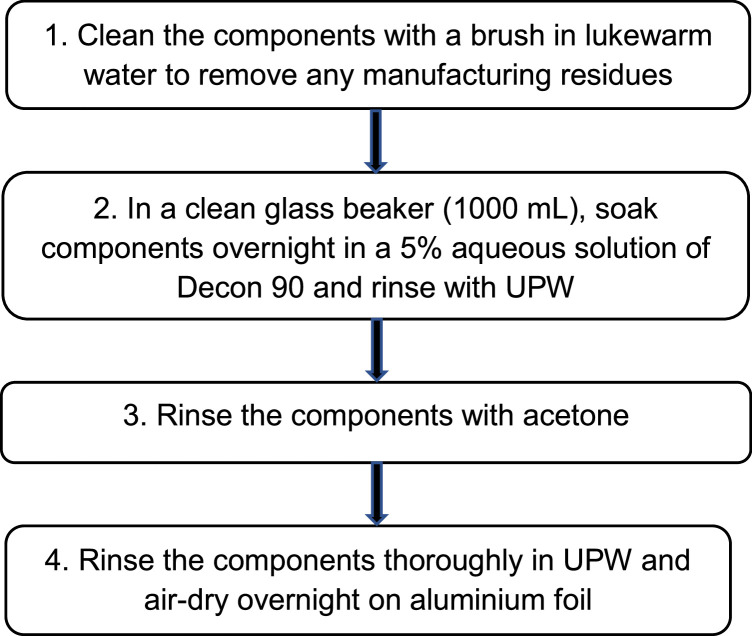

#### PES membrane preparation and cleaning

**Figure:**
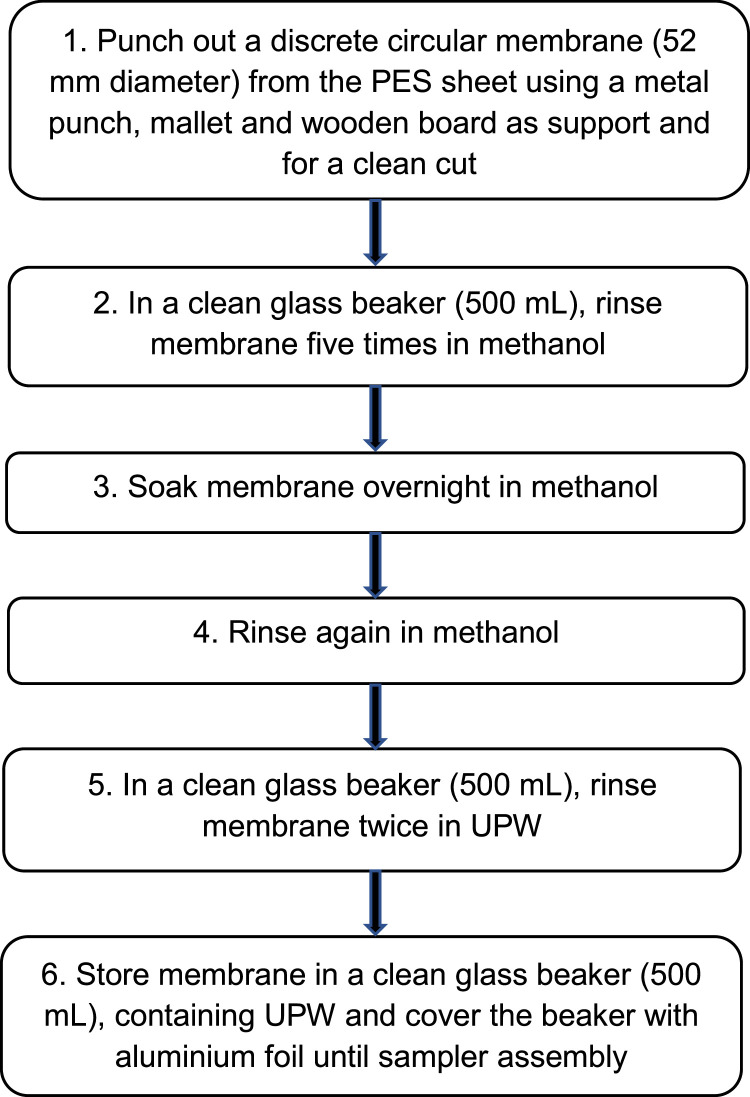

This step is essential to remove any residual polyethylene glycol that can remain as an artefact from the PES membrane manufacturing process [13]. Otherwise polyethylene glycol can be sequestered by the receiving phase disk during deployment. This can cause matrix/ion suppression effects during LC/MS analysis of the receiving phase extract.

#### Receiving phase disk cleaning and activation

**Figure:**
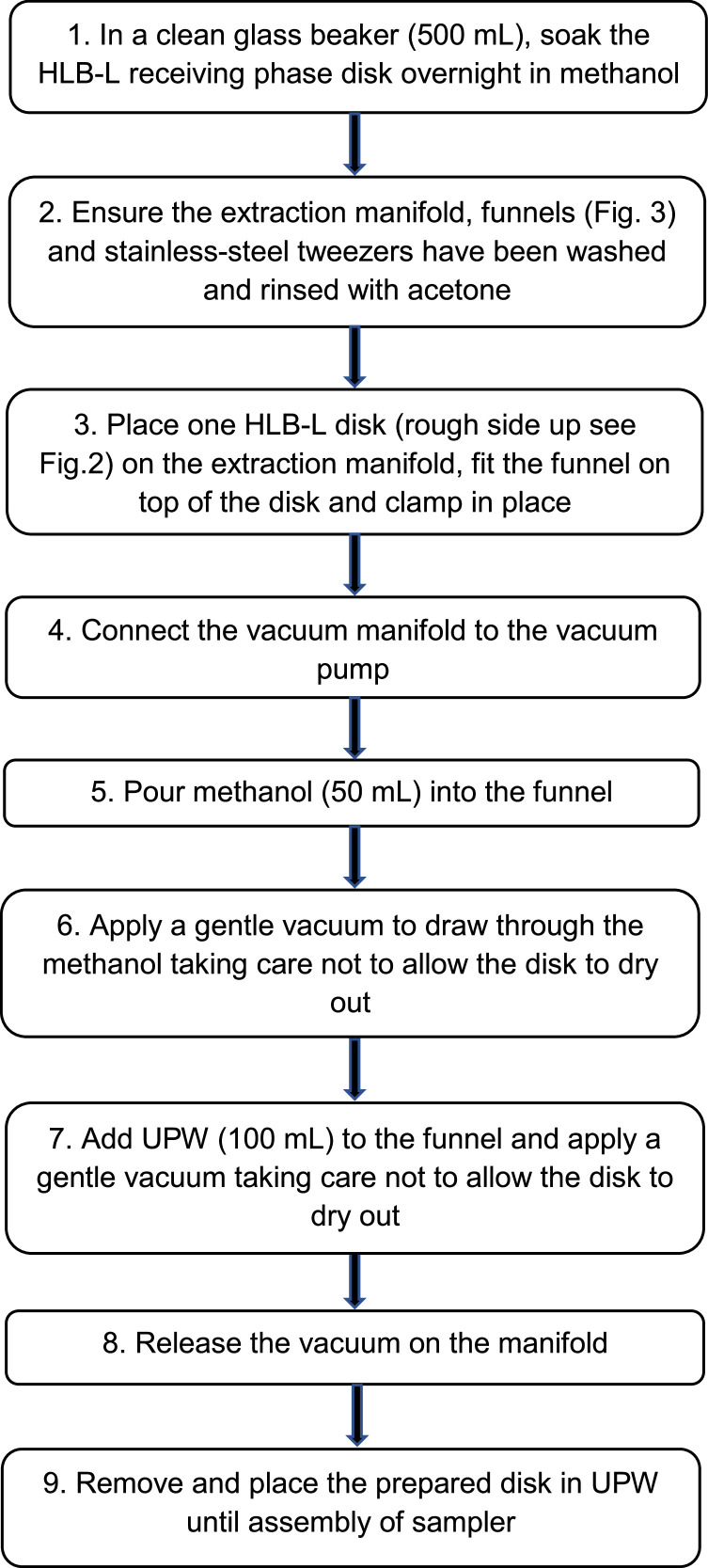

**Fig. 3:**
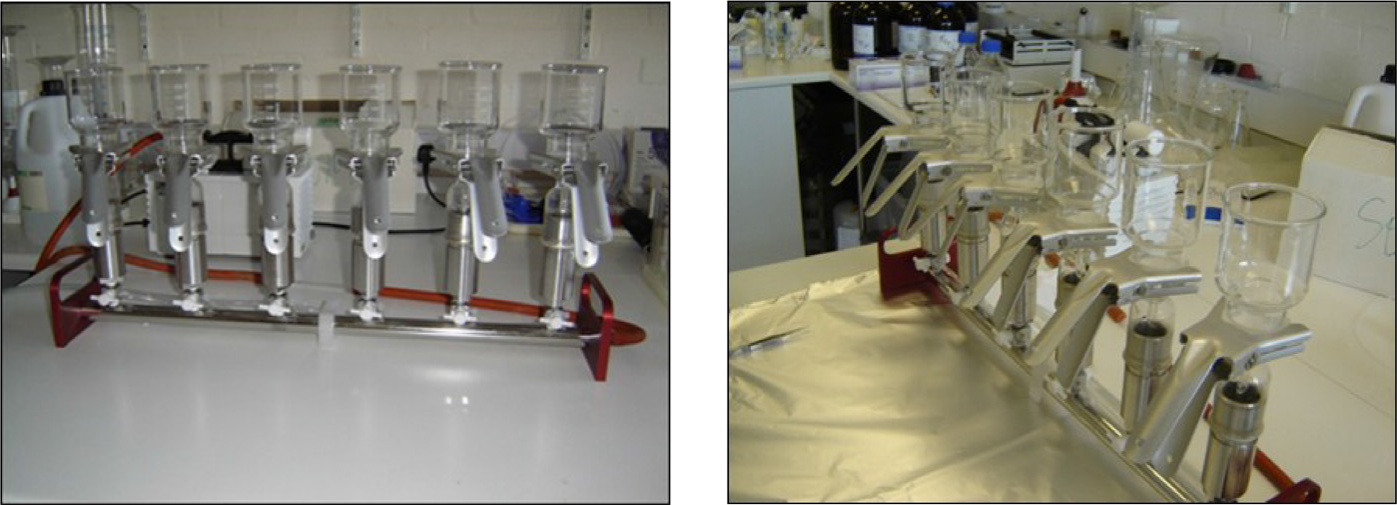
Six station manual glass vacuum manifold, showing base, funnels and clips.

#### Assembling the Chemcatcher device

**Figure:**
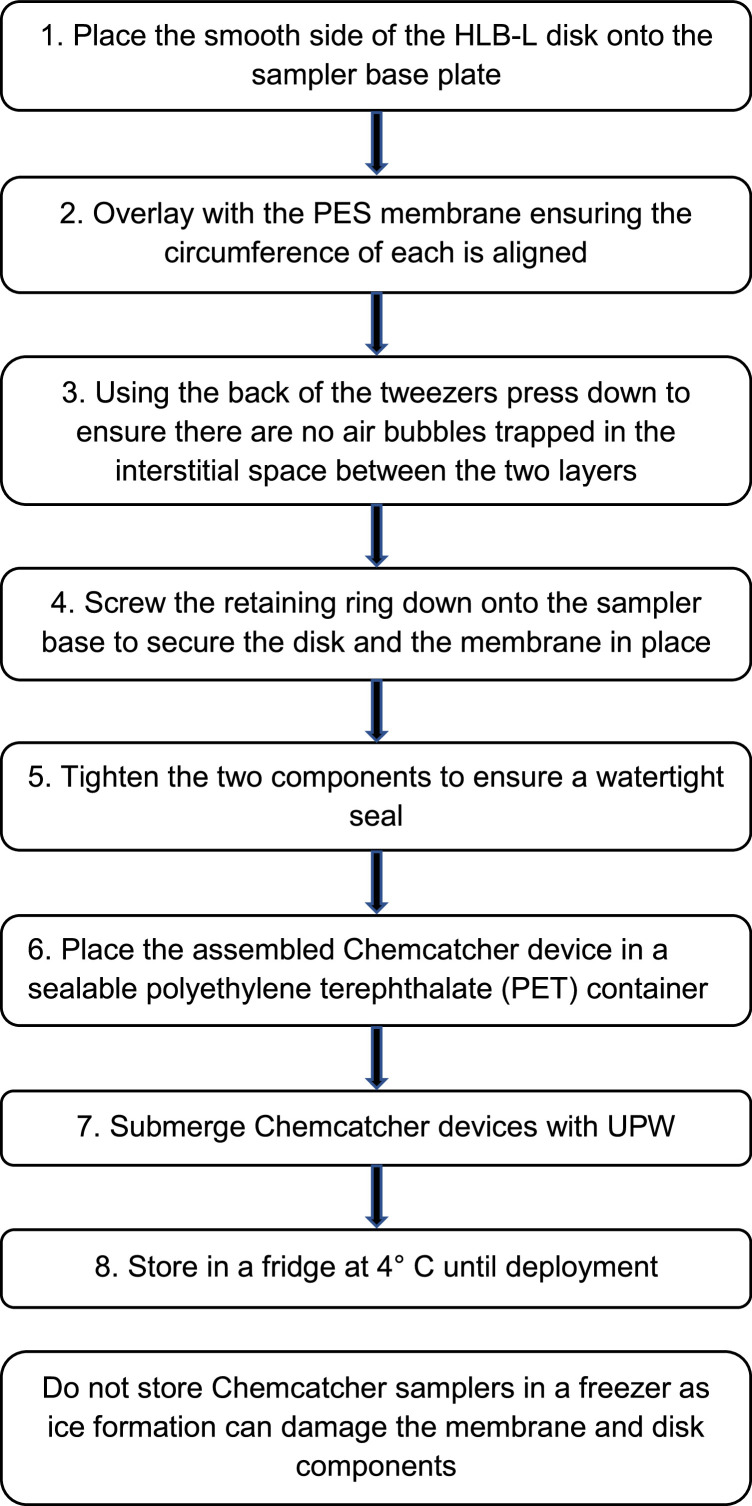

#### Chemcatcher quality control (QC)

Additional Chemcatcher samplers need to be prepared for quality control purposes.

#### Fabrication blank

The purpose of this QC sampler is to account for any background contamination by polar organic chemicals occurring during preparation, laboratory storage, processing and analytical procedures. Fabrication samplers are additional samplers, prepared together with the Chemcatcher samplers used in the field deployments. They are placed in a clean sealable PET box then submerged in UPW and stored at 4 °C in the laboratory until the extraction of the exposed samplers. Ideally three fabrication blanks should be used with each monitoring campaign.

#### Field blank

The purpose of this QC sampler is to account for any contaminants that accumulate in the Chemcatcher samplers during transportation and handling during deployment and retrieval at the field site/s. Field blank Chemcatcher samplers (stored in a clean sealable polyethylene terephthalate (PET) container and submerged in UPW) are transported to the field (in a cool box) together with the samplers to be exposed at the sampling location. Field blanks are exposed to the atmosphere during both the field deployment and retrieval periods of the active Chemcatcher samplers. After deployment the field blank samplers are stored at 4 °C in the laboratory. They are then taken back to the field deployment site/s when the active Chemcatcher samplers are retrieved. The field blank samplers are stored at 4 °C in the laboratory until extraction. Ideally, one field blank Chemcatcher sampler should be used at each sampling site.

### Chemcatcher deployment and retrieval

#### Transport to deployment site

Chemcatcher samplers are transported submerged in UPW in a sealable polyethylene terephthalate (PET) container. These are kept in a clean cool box with freezers blocks.

#### Deployment apparatus

Depending on field conditions different deployment apparatus is required. A number of different configurations are described in the literature [6], [7], [8], [9], [10]. It is important that the Chemcatcher samplers are deployed in flowing water, avoiding excessive turbulence. For this procedure we describe a generic deployment apparatus that is applicable for use at most riverine and other surface water sites with a relatively flat sediment r bed and for a riverine site a water depth of no more than ∼ 1 m. This system minimises the likelihood of fouling of vegetation/weed around the sampler and deployment apparatus.

• Concrete paving slab (12 mm diameter hole drilled in the centre)
• M12 stainless-steel eyebolt secured to the paving slab using two plain washers and a M12 nut
• Rope (∼ 1.5 m length) attached to the M12 eyebolt using a stainless-steel carabiner
• Stainless steel plate (made by AT Engineering - https://www.atengineeringtadley.co.uk/water-quality-monitoring/)
• Plastic mesh pocket

#### Deployment duration

Optimum deployment time depends on the dissolved concentrations of the analyte in water, the quantification limits of the analytical technique and the compound specific uptake rate into the sampler. Deployment times between 7 and 14 days are generally appropriate, providing that site-specific characteristics do not favor any significant biofouling development on the membrane sampling surface. At pristine sampling sites where pollutant concentrations are expected to be very low, then longer deployment periods may be more appropriate.

#### Number of sampler replicates

The number of samplers deployed on any one occasion depends on what questions are being answered by the monitoring campaign. For qualitative investigations it is best to deploy samplers in duplicate with one sampler being used as a backup or for archival purposes. For quantitative investigations, typically triplicate samplers are used at each deployment site.

#### Health and safety

A suitable risk assessment must be prepared detailing the possible hazards such as working in rivers, hygiene and the mitigation strategies required in accordance with establishment's protocol.

#### Site security

Access to samplings sites in rivers may require explicit permission from riparian owners. Security of each site must be maintained such as closing and padlocking gates.

#### Chemcatcher deployment

**Figure:**
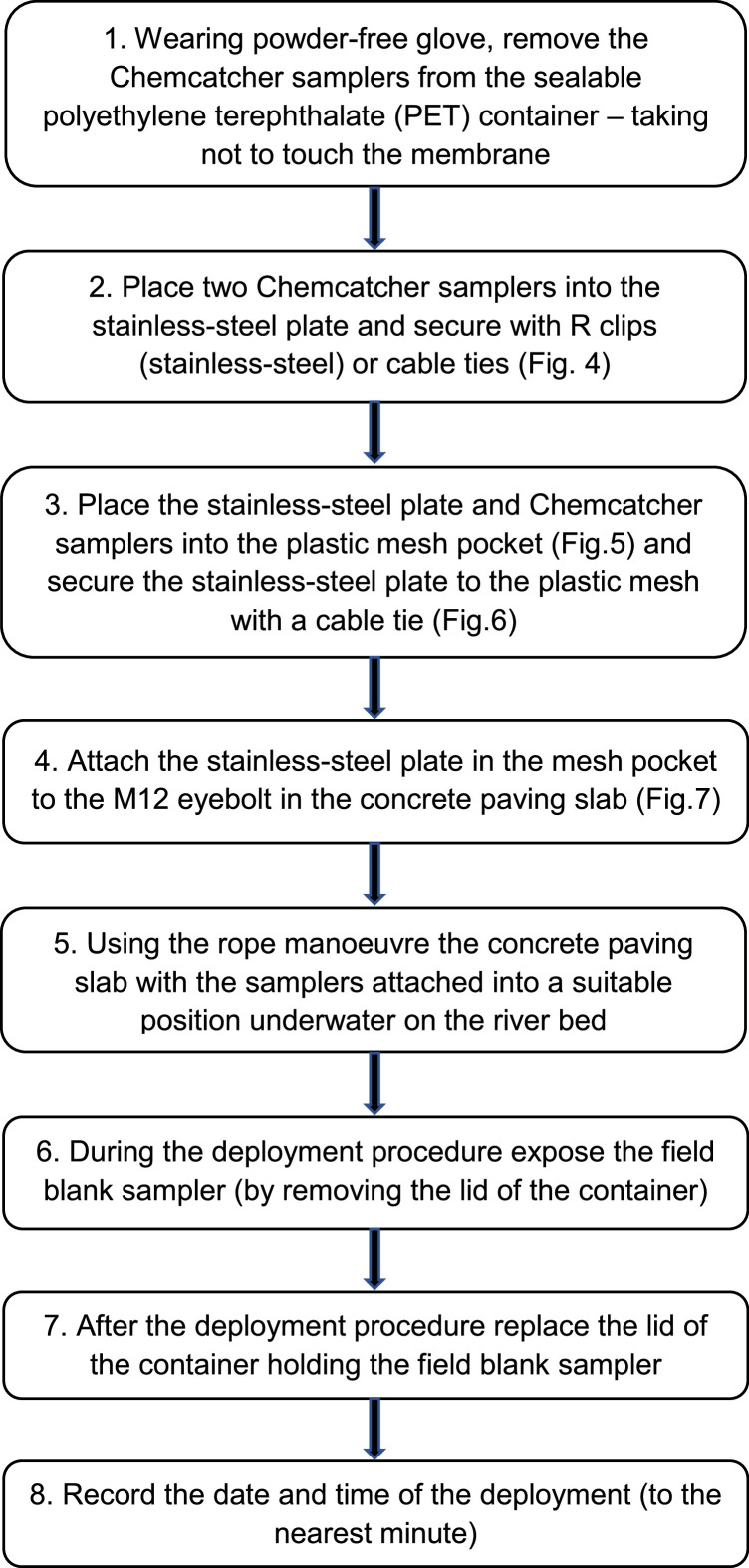

**Fig. 4:**
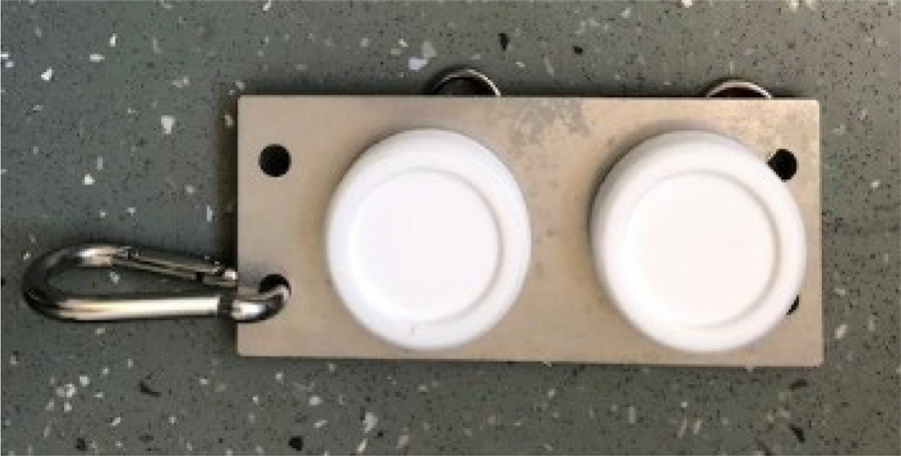
Chemcatcher devices attached to the stainless-steel plate using R clips.

**Fig. 5:**
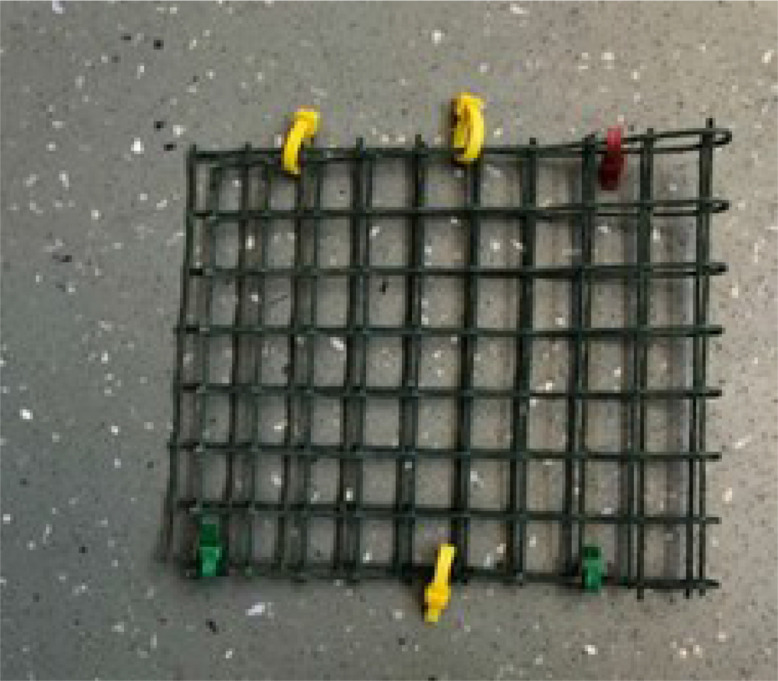
Plastic mesh pocket.

**Fig. 6:**
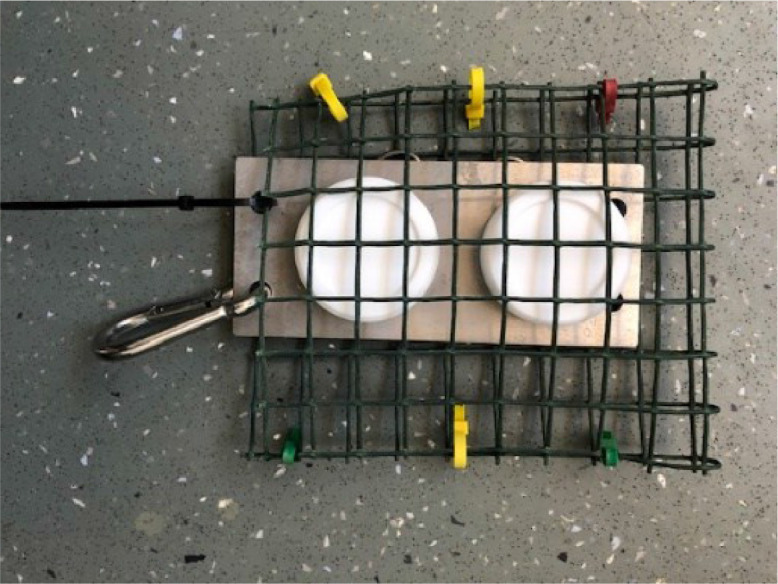
Chemcatcher samplers placed inside the plastic mesh pocket.

**Fig. 7:**
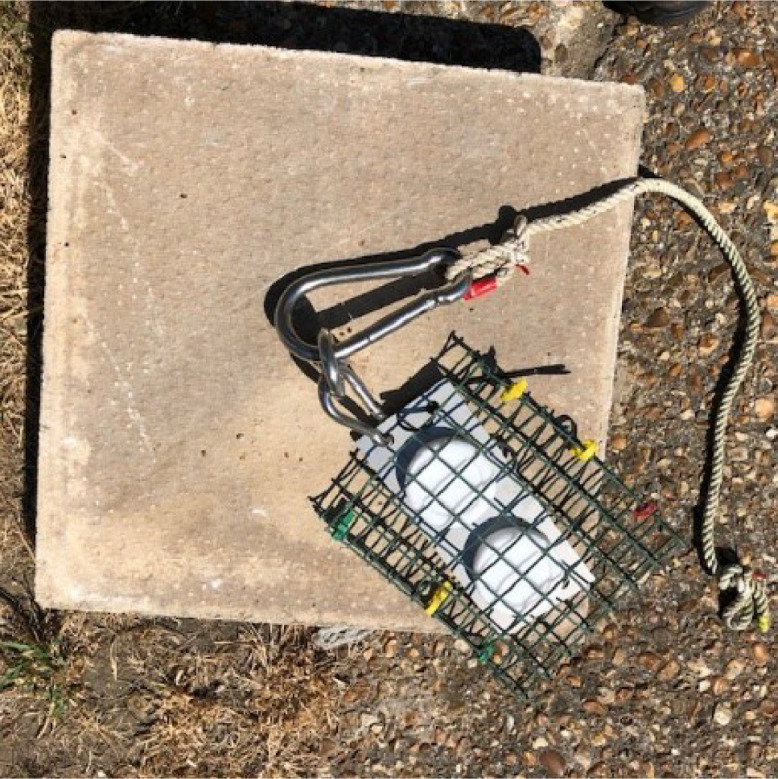
Stainless steel plate attached to the deployment concrete slab and deployment rope attached to eye bolt.

#### Chemcatcher retrieval

**Figure:**
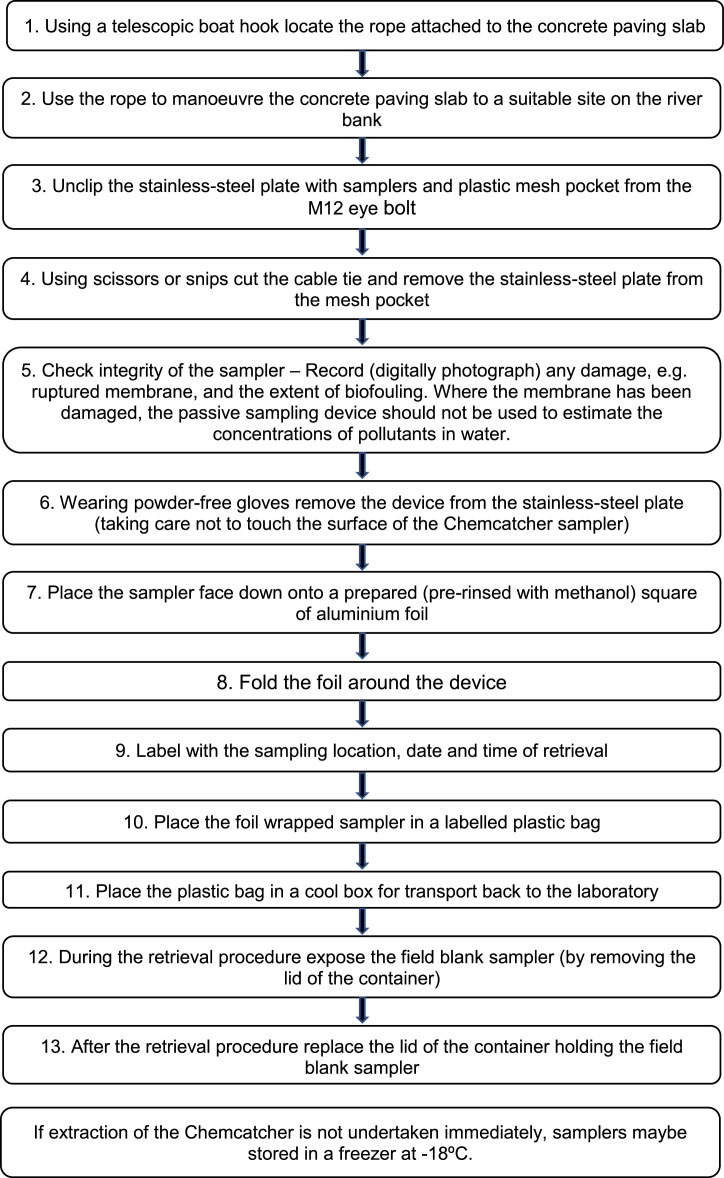

### Water quality parameters and sampling site measurements

Water quality parameters can also be measured when the Chemcatcher sampler is deployed and retrieved. The most frequently measured parameters include: depth, temperature (°C), conductivity, dissolved oxygen (% and mg L^−1^) and pH. These are normally taken using a calibrated multi-sonde instrument (e.g. YSI EXO sonde) at the depth of the deployment of the Chemcatcher. Individual parameter probes can also be used if a multi-sonde instrument is not available.

Average (60 s) flow rate (m s^−1^) measurements are also taken when the Chemcatcher is deployed and retrieved. This is normally undertaken using a digital flowmeter (e.g. Valeport flowmeter). If discharge (m^3^ s^−1^) is needed then the river profile (cross sectional area in m^2^) needs to be ascertained. This can be achieved using surveyors’ tape measure and water depth pole. Ideally, the depth needs to be measured at 1 m intervals.

### Chemcatcher receiving phase disk extraction

The processing needs to be undertaken in laboratory suitable for trace organic analysis. The sequence for dismantling Chemcatcher samplers should follow from the least to the highest expected contamination: (i) fabrication blank, (ii) field blank and (iii) deployed samplers.

**Figure:**
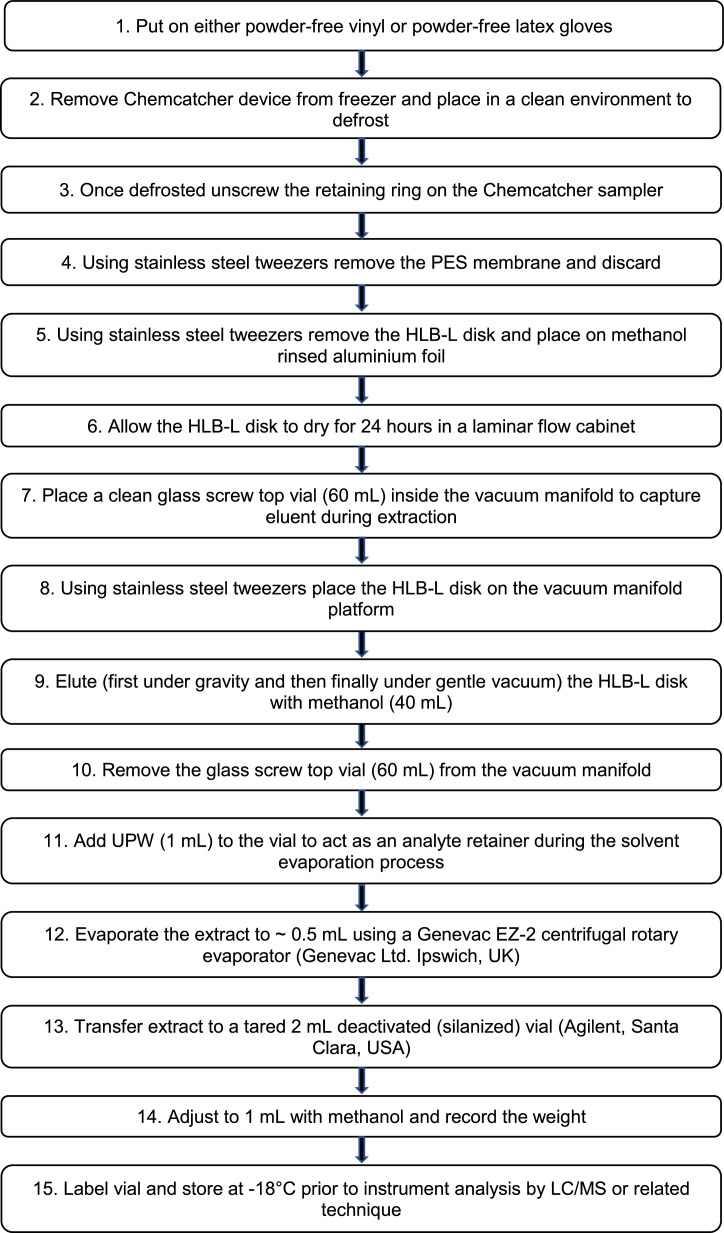

#### Conclusions

This protocol provides standard methods and guidelines for the laboratory preparation of Chemcatcher passive samplers for the measurement of polar organic compounds. It includes information on the preparation of deployment equipment suitable for monitoring pollutants in surface water. Additionally, details are given on the extraction of sequestered analytes on the receiving phase disk up to the point of instrumental analysis. This protocol has been used successfully to monitor a wide range of polar pollutants (such as plant protection products, pharmaceuticals and personal care products) in rivers and streams worldwide.

## CRediT authorship contribution statement

**Rosamund F.A. Robinson:** Writing – original draft, Validation. **Graham A. Mills:** Conceptualization, Methodology, Writing – review & editing. **Gary R. Fones:** Methodology, Resources, Writing – review & editing, Supervision.

## Declaration of Competing Interest

The authors declare that they have no known competing financial interests or personal relationships that could have appeared to influence the work reported in this paper.

## Data Availability

No data was used for the research described in the article.
